# Comparison of the quality of basic life support provided by rescuers trained using the 2005 or 2010 ERC guidelines

**DOI:** 10.1186/1757-7241-20-53

**Published:** 2012-08-09

**Authors:** Christopher M Jones, Andrew Owen, Christopher J Thorne, Jonathan Hulme

**Affiliations:** 1 Resuscitation for Medical Disciplines, College of Medical & Dental Sciences, University of Birmingham, Birmingham, B15 2TT, UK; 2 Queen Elizabeth Hospital Birmingham, University Hospitals Birmingham NHS Foundation Trust, Birmingham, B15 2WB, UK; 3 Sandwell and West Birmingham Hospitals NHS Trust, City Hospital, Dudley Road, Birmingham, B18 7QH, UK

**Keywords:** Adult, Basic life support (BLS), Cardiopulmonary resuscitation (CPR), 2005 European Resuscitation Council (ERC) guidelines, 2010 European Resuscitation Council (ERC) guidelines, Cardiac arrest

## Abstract

**Introduction:**

Effective delivery of cardiopulmonary resuscitation (CPR) and prompt defibrillation following sudden cardiac arrest (SCA) is vital. Updated guidelines for adult basic life support (BLS) were published in 2010 by the European Resuscitation Council (ERC) in an effort to improve survival following SCA. There has been little assessment of the ability of rescuers to meet the standards outlined within these new guidelines.

**Methods:**

We conducted a retrospective analysis of the performance of first year healthcare students trained and assessed using either the new 2010 ERC guidelines or their 2005 predecessor, within the University of Birmingham, United Kingdom. All students were trained as lay rescuers during a standardised eight hour ERC-accredited adult BLS course.

**Results:**

We analysed the examination records of 1091 students. Of these, 561 were trained and assessed using the old 2005 ERC guidelines and 530 using the new 2010 guidelines. A significantly greater proportion of candidates failed in the new guideline group (16.04% vs. 11.05%; p < 0.05), reflecting a significantly greater proportion of lay-rescuers performing chest compressions at too fast a rate when trained and assessed with the 2010 rather than 2005 guidelines (6.04% vs. 2.67%; p < 0.05). Error rates for other skills did not differ between guideline groups.

**Conclusions:**

The new ERC guidelines lead to a greater proportion of lay rescuers performing chest compressions at an erroneously fast rate and may therefore worsen BLS efficacy. Additional study is required in order to define the clinical impact of compressions performed to a greater depth and at too fast a rate.

## Background

Sudden cardiac arrest (SCA) is a significant cause of mortality within Europe and the United States [1-3]. The incidence of out-of-hospital SCA is estimated to be between 0.4 and 1 per 1000 in Europe, contributing to a high disease burden [3]. Although the outcome of SCA is dependent on a number of intrinsic variables, survival is largely reliant on prompt and effective cardiopulmonary resuscitation (CPR) and defibrillation [4-6].

Given the importance of intervention to SCA outcome, standards and guidelines for CPR have been used to train prospective rescuers for over 30 years [7]. Despite the availability and continued development of these guidelines, survival from out-of-hospital SCA remains relatively low [1,2,8]. This may be attributable, at least in part, to rates of community CPR remaining static since the 1970s [9]. In addition, rescuers’ CPR performance is not routinely measured and little is known about the degree to which its quality differs to that outlined by consensus guidelines [10].

Recommended standards for response to SCA are routinely updated and the European Resuscitation Council (ERC) published its most recent guidelines for adult basic life support (BLS) in 2010 [11]. In comparison to their 2005 predecessor, the 2010 ERC guidelines for adult BLS advise that chest compressions are performed to a greater depth (5-6 cm compared to the previously advised depth of 4-5 cm) and within a more closely defined range of 100–120 compressions per minute (previously stipulated to be around 100 compressions per minute, but assessed on ERC BLS 2005 assessment forms as 80–120 compressions per minute) [11,12].

Very little is known about the impact of the 2010 guidelines on the quality of CPR delivered by rescuers. In addition, a paucity of research has been conducted to determine the degree to which rescuers are able to comply with guideline changes, under simulated conditions.

This study will therefore assess the performance of lay rescuers during assessment of their BLS skills when using the 2010 ERC guidelines compared to the 2005 guidelines.

## Materials and methods

### Study design

We conducted a retrospective observational study of the performance of first year University of Birmingham healthcare students trained and assessed using either the 2005 or 2010 ERC adult BLS guidelines. All records for examinations undertaken within our course over a period of two academic years, extending from October 2009 to April 2011, were included in the analysis. No records from assessments taken within the designated time period were omitted from the analysis.

### Population & setting

As has been previously described, first year preclinical medicine, dentistry and physiotherapy students receive innovative peer-led BLS tuition within our institution [13,14]. Due to their lack of clinical knowledge and experience, all are taught and assessed as lay-rescuers. This successful programme, utilising senior healthcare students to instruct their junior colleagues, follows a standardised eight hour syllabus followed by summative assessment under examination conditions. All teachers are trained as ERC BLS/AED instructors and standardised tuition is delivered in small groups (at a maximum trainer-to-tutor ratio of 3:1) using the four-stage approach [15]. Students are trained in accordance with the latest ERC guidelines and supervision of this training is carried out by a faculty of senior students and doctors, for quality assurance purposes.

In our training programme, candidates’ ability to correctly perform BLS on a Resusci® Anne Basic manikin (Laerdal Medical Limited, Orpington, Kent, UK) is observed at the end of their course by an assessor, at a 1:1 ratio. A standardised sequential list of skills must be achieved during this examination by prospective lay-rescuers (the marking sheets used for the 2005 and 2010 criteria are shown in Additional file 1: Figure S1). These are based on ERC guidelines and provide an overview of accepted performance on which the assessor can base their final decision on whether to pass or fail a candidate.

Students trained and assessed using either the 2005 or 2010 guidelines were, for example, taught to maintain a rate of between 80–120 compressions per minute and 100–120 compressions per minute, respectively. They failed their assessment if compressions were not completed within this designated range. Rate was measured by assessors utilising a stopwatch in both assessment years and students were expected to complete a cycle of 30 chest compressions within a defined time period of 15–22.5 seconds and 15–18 seconds when taking assessments under the 2005 and 2010 guidelines respectively, equivalent to 80–120 and 100–120 compressions per minute.

All assessors within our course have achieved an ERC-accredited provider and instructor qualification and receive additional in-house assessor training, the format of which did not vary between the two years in question. Approximately 50% of the assessor cadre remained constant throughout the two sampled years. Senior external examiners were used to audit and standardise assessor performance, as is routine for our course.

### Outcome measures

Overall failure rates were compared between the 2005 and 2010 ERC guideline groups, in addition to the following dependent variables: whether rescuer called for help, effective airway management, breathing assessment, evidence of call to emergency services, successful rescue breath delivery, correct ventilation : compression ratio and accuracy of chest compression hand position, depth and rate.

### Statistical analysis

A two tailed chi-squared test was used for all statistical comparisons. p-values of less than 0.05 were considered significant.

## Results

The assessment records of 1091 students were compared: 561 were trained and assessed using the old 2005 ERC guidelines (during the 2009–2010 academic year) and 530 using the new 2010 ERC guidelines (during the 2010–2011 academic year). The comparability of the two groups is outlined in Table 1; the 2005 guideline group featured a marginally higher proportion of medicine and physiotherapy students than the 2010 group (75.2% and 13.0% compared with 75.0% and 11.5% respectively). In addition, males constituted an additional 7.2% of the studied population for the 2005 guideline group when compared with those following 2010 recommendations. Mean ages were similar at 19.4 ^+^/_−_ 2.0 years and 19.2 ^+^/_−_ 1.1 years for the 2005 and 2010 guideline groups respectively.

**Table 1:** Summary of study participants’ demographic data

**Academic year analysed**	**Age (years old)**		**Gender (%)**		**Course (%)**		
	**Mean**	**SD**	**Male**	**Female**	**Medicine**	**Dentistry**	**Physiotherapy**
2009 - 2010	19.4	2.0	38.4	61.6	75.2	11.8	13.0
2010 - 2011	19.2	1.1	31.2	68.8	75.0	13.5	11.5

Overall performance of BLS was significantly worse using the new guidelines, as shown in Table 2: 16.0% failed their assessment (i.e. unsuccessfully completed one or more criteria) compared to 11.1% in the old group (p < 0.05). There is, in addition, a disparity in the total number of skills each rescuer performed unsuccessfully. For instance, a significantly greater proportion of rescuers in the new guideline group failed their examination due to unsuccessfully performing one skill only (11.9% vs. 8.0%; p < 0.05). Yet, in contrast, the percentage of lay-rescuers inadequately performing two or more skills does not significantly differ between old and new guideline groups (3.1% compared to 4.2% respectively (p > 0.05)).

**Table 2:** Summary of differences in BLS errors between rescuers trained with 2005 and 2010 ERC guidelines

**Study overview**					
Total number assessed using 2005 ERC guidelines during 2009–2010 academic year:					561
Total number assessed using 2010 guidelines during 2010–2011 academic year:					530
Total number assessed over both studies academic years:					1091
**Overall error rates**					
Number of errors made per rescuer	2005 Guidelines		2010 Guidelines		p-Value
	No. of rescuers	Percentage of rescuers	No. of rescuers	Percentage of rescuers	
No error	499	88.9	445	84.0	0.016
One error	45	8.0	63	11.9	0.033
Two errors	15	2.7	20	3.8	0.303
Three errors	2	0.4	2	0.4	0.955
**Error rates per skill**					
Skill	2005 Guidelines		2010 Guidelines		p-Value
	No. of rescuers	Percentage of rescuers	No. of rescuers	Percentage of rescuers	
Follows algorithm in correct sequence	0	0.0	2	0.4	0.145
Call for help	0	0.0	2	0.4	0.145
Open airway	7	1.3	12	2.3	0.200
Assess breathing	1	0.2	0	0.0	0.331
Telephone for help	1	0.2	0	0.0	0.331
Chest compression hand position	8	1.4	11	2.1	0.412
Chest compressions of insufficient depth	4	0.7	5	0.9	0.674
Chest compression rate too slow	22	3.9	13	2.5	0.169
Chest compression rate too fast	15	2.7	32	6.0	0.006
Number of compressions delivered	0	0.0	2	0.4	0.145
Ventilation-compression ratio	22	3.9	30	5.7	0.178

A significantly greater proportion of lay-rescuers performed chest compressions at too fast a rate in the new 2010 guideline group than in the old (6.04% compared with 2.67%; p < 0.05). There was no significant difference in the number of rescuers performing chest compressions too slowly (2.45% new, 3.92% old; p > 0.05). Use of the new guidelines did not significantly alter the proportion of candidates erroneously performing other assessed skills.

## Discussion

The new 2010 ERC life support guidelines stress the importance of high quality chest compressions for effective BLS, and advise that they are performed to a greater depth and within a narrower range than recommended by their 2005 predecessor [11,12].

The importance of maintaining a compression rate above 100 min^-1^ is emphasised within the new guidelines but the upper limit of 120 min^-1^ remains. Despite this, the rate of chest compression delivery was too fast in a significantly increased fraction of rescuers trained with the 2010, rather than 2005, guidelines. No significant difference in rescuers’ ability to perform any of the other tested skills without error is incurred with use of the new guidelines.

A significantly greater proportion of lay-rescuers trained with the new ERC guidelines performed at least one skill erroneously when demonstrating BLS. The overall increase seen in lay-rescuers performing BLS erroneously therefore appears to be directly related to rescuers completing chest compressions at too fast a rate due to an inability to meet the narrower confines for rate recommended by the new ERC guidelines.

This may result from course teachers emphasising the need to perform chest compressions at a relatively faster rate that does not fall to the lower limit of 80–99 compressions minute^-1^ that was permitted by previous guidelines. In addition, the need to perform chest compressions at a greater depth may instil within CPR providers a mentality of ‘harder and faster’, through which the more energetic pursuit of greater chest compression depth results in a faster rate.

In order to explore this latter point, further research is required to determine whether lay providers are able to skilfully and effectively dissociate deep chest compressions from a tightly controlled rate of chest compression delivery. In addition, basic life support course directors and instructors should be reminded to focus training for providers towards delivering chest compressions at the depth required by the 2010 guidelines, whilst within a controlled rate (with the use of a metronome, for instance, rather than adopting and disseminating a ‘harder and faster’ mentality towards the new ERC guidelines).

The effect of providers seemingly adopting this mentality is, however, unclear. There exists a strong positive correlation between the number of chest compressions delivered to a casualty each minute and the likelihood of successful outcome [16]. However, although faster chest compressions are thought to improve prognosis by generating greater blood flow to the brain and myocardium, animal studies have indicated that cardiac output reaches a plateau between 60 to 120 compressions per minute [17-19].

At rates of greater than 120 compressions per minute, available time for both diastolic myocardial perfusion and adequate venous return decreases [17-19], thereby potentially limiting coronary perfusion rates and decreasing cardiac output. Furthermore, fatigue occurs earlier, with greater impact on CPR quality, if the number of chest compressions delivered by lay-rescuers is increased [20]. Lay rescuers are unable to meet the narrower range for chest compression rate recommended within the new 2010 ERC life support guidelines in our study. Their resultant tendency to complete chest compressions at rates greater than 120 compressions per minute may therefore decrease the likelihood of successful CPR following cardiac arrest through reduced blood flow to vital organs and increased rescuer fatigue.

It is, however, difficult to detail precisely the potential clinical impact of our findings. For example, this study is limited in its inability to specify the degree to which chest compression rate exceeded the recommended upper maximum. In addition, rescuers using the 2005 and the 2010 guidelines performed all other assessed skills without significant apparent variation in error rates. Lay rescuers were therefore seemingly able to perform compressions at the greater depth required by the new guidelines as successfully as those meeting a shallower depth in the old guidelines. This measurement was subjective, however, and assessors’ appraisal of the depth of trainees’ chest compressions may have been inaccurate as a result. Nevertheless, the effect of deeper chest compressions performed at too fast a rate is not fully known and may require elucidation, particularly if future studies confirm the objective data outlined within this report.

Despite these limitations, the peer-led course from which the data for this study is sourced has run successfully for over 15 years and is of high repute. Instructors are trained to meet international standards, and deliver teaching in accordance with recommendations from the ERC and to the same standard as external faculty [14]. For the periods from which the data included in this study is taken, teaching standards were rigorously reviewed by the same experienced external faculty members for similar periods of time. Therefore, although we acknowledge that our instructors are not under constant supervision, teaching quality is unlikely to have varied within or between courses due to the measures taken in order to ensure standardisation.

End of course assessments on which this study was based were carried out similarly for prospective rescuers using the 2005 and 2010 guidelines. Senior students assessed candidates using set written criteria (which varied only to reflect changes in ERC guidelines, as shown in Additional file 1: Figure S1) and were moderated by external faculty. We have previously shown that agreement between external faculty and student observers is high and do not envisage that the results of this study were affected by the nature of candidates’ assessment [14].

## Conclusions

The new 2010 ERC adult life support guidelines are more difficult to perform than their 2005 predecessor, leading to a greater proportion of lay rescuers completing chest compressions at too fast a rate. Further study is required to support this finding and assess its impact on outcome following SCA.

## Abbreviations

SCA, Sudden cardiac arrest; ERC, European resuscitation council; BLS, Basic life support; CPR, Cardiopulmonary resuscitation; AED, Automated external defibrillator.

## Competing interests

The authors declare that they have no competing interests.

## Authors’ contributions

CMJ, AO, CJT and JH jointly conceived and designed the study. Data was routinely collected and subsequently collated by CMJ and CJT. CMJ, AO and CJT contributed to initial analysis of data, with all authors contributing to data interpretation. CMJ and AO drafted the manuscript. CMJ, AO, CJT and JH critically read the manuscript, and all made significant contributions to revisions. All authors have read and approved the final manuscript.

## Supplementary Material

Additional file 1: Figure S1 Mark sheet detailing pass criteria for candidates trained and assessed using 2005 and 2010 ERC guidelines. Click here for file
