# Supplementary figures and images for: Comparison of the quality of basic life support provided by rescuers trained using the 2005 or 2010 ERC guidelines
Source: Scand J Trauma Resusc Emerg Med. 2012 Aug 9;20:53. doi: 10.1186/1757-7241-20-53 (PMC3462103; doi:10.1186/1757-7241-20-53)

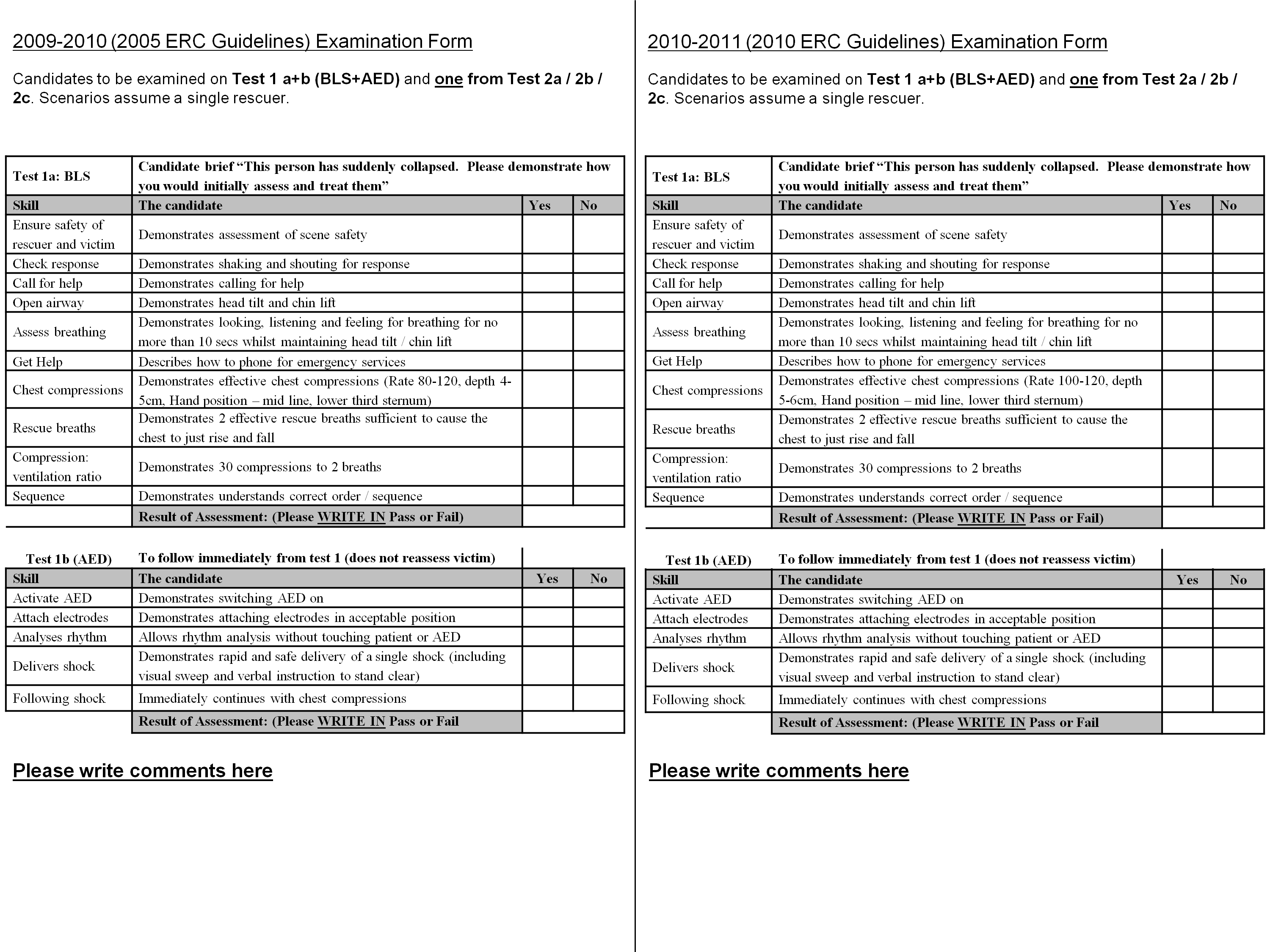

Supplement: Additional file 1: Figure S1 — Mark sheet detailing pass criteria for candidates trained and assessed using 2005 and 2010 ERC guidelines. [file 1757-7241-20-53-S1.tiff]
